# The co-expression of CBX8 and PD-L1 and prognostic value in cervical cancer

**DOI:** 10.1097/MD.0000000000027056

**Published:** 2021-08-27

**Authors:** Hui Zhou, Anhong Li, Chuan Li, Mingyong Wu, Dan Jin, Mingcai Shui

**Affiliations:** Department of Oncology, The People's Hospital of Kaizhou District, Kaizhou, Chongqing, China.

**Keywords:** chromobox homolog 8, cervical cancer, programmed death ligand-1, prognosis

## Abstract

Chromobox homolog 8 (CBX8) plays an important role in the occurrence and development of various tumors, and is closely related to the prognosis of patients with cancer. However, the occurrence, development, and prognostic value of CBX8 in cervical cancer have not been reported yet.

In this study, immunohistochemistry was used to detect the expression of CBX8 in cervical cancer tissues and the corresponding normal tissues adjacent to the tumor. Furthermore, the relationship between CBX8 and programmed death-ligand 1 (PD-L1) expression, clinicopathological characteristics, and prognosis of cervical cancer were explored, and the prognostic value of CBX8 in cervical cancer was clarified.

In this study, the results of immunohistochemistry using tissue chips obtained from patients with cervical cancer showed that CBX8 was highly expressed in cervical cancer tissues, and its expression was proportional to the international federation of gynecology and obstetrics (FIGO) stage. Disease-free and overall survival of patients with high CBX8 expression in cervical cancer were significantly shorter than those of patients with low CBX8 expression. Thus, CBX8 was found to be an independent prognostic factor for patients with cervical cancer. In addition, CBX8 and PD-L1 co-expression model could better predict the prognosis of patients with cervical cancer, and its area under the receiver operating characteristic curve was similar to that of FIGO stage.

CBX8 may be an independent prognostic factor for cervical cancer. Moreover, the CBX8 and PD-L1 co-expression model could predict the postoperative survival of patients with cervical cancer objectively and reliably, which will aid clinicians to shunt patients with cervical cancer based on the risk of death, develop a reasonable treatment plan, and provide personalized prognosis.

## 1 Introduction

Cervical cancer is one of the most common malignancies in women in the world and seriously threatens the long-term health of women. According to the latest global cancer annual report, there were approximately 569,847 new cases of cervical cancer worldwide in 2018, and the number of deaths of cervical cancer reached 311,365.^[1]^ In recent decades, owing to the popularization of cancer screening and the introduction of human papillomavirus infection (HPV) vaccines, the incidence rate of cervical cancer has shown a downward trend in many countries.^[2]^ However, the incidence and mortality rates of cervical cancer greatly vary with the geographical location and economic conditions in various regions. A study in 38 countries and 5 continents showed a steady or even rising trend in the incidence of cervical cancer in developing countries with a declining trend in developed countries.^[3]^ The latest National Cancer Statistics released by National Central Cancer Registry of China in 2015 showed that cervical cancer ranks sixth among the top 10 female malignant tumors.^[4]^ Currently, treatment methods of cervical cancer are being developed slowly. Surgery, chemotherapy, and radiotherapy are still the main treatment methods for cervical cancer patients,^[2,5]^ and the efficacy of targeted therapy is limited. Therefore, identifying novel diagnostic and therapeutic targets for cervical cancer patients and exploring targeted therapeutic drugs with definite curative effects have great significance in the early diagnosis and improvement of cervical cancer prognosis.

Polycomb group (PcG) proteins found in *Drosophila* are transcription factors that can modify and regulate target genes at the chromatin level. Its main function is to inhibit and silence target gene transcription, and its abnormal state can lead to the occurrence of cancer directly or indirectly.^[6]^ Chromobox homolog (CBX) protein is an important member of the PcG family, mainly including 5 homologs: CBX2, CBX4, CBX6, CBX7, and CBX8, all of which contain highly conserved chromatin domains and Pc boxes, but their functions are different.^[7]^ It has been found that CBX8 plays an important role in the occurrence and development of various tumors,^[8–10]^ and is closely related to the prognosis of patients with cancer, including colorectal and esophageal cancers, and hepatocellular carcinoma.^[11–13]^ However, the occurrence, development, and prognostic value of CBX8 in cervical cancer have not been reported yet. Currently, there are no accurate and reliable indices that are routinely used for the prognostic evaluation of gastric adenocarcinoma. In this study, we aimed to analyze the expression of CBX8 in cervical cancer tissues and corresponding normal tissues adjacent to the tumor and investigate the relationship between CBX8 and programmed death-ligand 1 (PD-L1) expression, clinicopathological characteristics, and prognosis of patients with cervical cancer to clarify the impact of CBX8 on cervical cancer, improve the survival rate and quality of life of patients, and provide a new target for the treatment of cervical cancer.

## 2 Methods

### 2.1 Cervical cancer tissue chip and matched case data

Cervical cancer tissue chips, including HUteS169Su01, OD-CT-RpUtr03–004, OD-CT-RpUtr03–005, and OD-CT-RpUtr03–006, were provided by Shanghai Outdo Biotech. OD-CT-RpUtr03–004, OD-CT-RpUtr03–005 and OD-CT-RpUtr03–006 contained a total of 93 cases of cervical cancer tissues and corresponding paracarcinoma tissues. HUteS169Su01 contained 126 cases of cervical cancer tissues, with complete clinicopathological data and follow-up data. All patients underwent radical resection of cervical cancer from January 2010 to October 2011. None of them received anti-tumor treatment before surgery. They were confirmed to be diagnosed with cervical squamous cell carcinoma by pathologists. The follow-up period started from the date of the patient's operation until March 2017 (if the patient died during the period, the time of death was the end of follow-up). The median follow-up time was 75 months (range: 8–86 months). Shanghai Outdo Biotech officially declares that sample collection of all clinical specimens has been approved by the patients, and the patients provided written informed consent prior to sample collection. This study adheres to the Declaration of Helsinki and the study protocol was reviewed and approved by the Ethics Committee of the People's Hospital of Kaizhou District.

### 2.2 Immunohistochemistry

The cervical cancer tissue chips were baked horizontally and vertically in an oven at 85°C for 60 minutes, followed by dewaxing, debenzene, and hydration. After antigen retrieval, they were washed thrice with phosphate-buffered saline (PBS), immersed in H_2_O_2_ solution at a concentration of 3% for 30 minutes, and then blocked with antigen. Bovine serum albumin (0.3% BSA: 0.15 g BSA + 5 mL double-distilled water) was prepared, and 200 μL of BSA was added to the surface of the tissue chips, a circle was drawn around the tissue with an immunohistochemical pen, and air bubbles were repeatedly removed with the tip of the gun. The incubation box was taken out, and the tissue chip with BSA was put into it at 37°C for 30 minutes. With the primary antibody (total number and volume of chips) prepared, the ratio was calculated. The blocking solution on the chip was removed and the primary antibody was added directly at 4°C overnight. After the primary antibody was discarded, the chips were washed thrice with PBS (5 minutes each time). The secondary antibody was selected according to the source of the primary antibody. Subsequently, the chips were washed thrice with PBS (5 minutes each time); 3,3′-Diaminobenzidine developer was prepared: 1 drop of 850 μL deionized water + A + B + developer, and they were shaken evenly until the yellow particles were observed under the microscope. The chips were then washed thrice with PBS (5 minutes each time). The stained chips were transferred to the hematoxylin shelf for hematoxylin counterstaining for 5 to 10 second, followed by blue returning in cold water tank for 5 to 10 minutes. After washing, the chips were dehydrated and mounted.

Twenty visual fields were randomly selected to observe tissue sections under a light microscope (10 × , 100 × , 200 × , and 400 × ) by 2 pathologists who were double-blinded. The histo (H) score was used for evaluation. Dark brown dots indicated strongly positive, brown-yellow dots indicated moderately positive, light yellow dots indicated weakly positive, and blue nuclei indicated negative. The percentage of areas with different staining (strong, moderate, weak, negative) was analyzed in pixels. Finally, the H score was calculated using the following equation: (percentage of negatively stained area × 0) + (percentage of weakly stained tumor cells × 1) + (percentage of moderately stained tumor cells × 2) + (percentage of strongly stained tumor cells × 3).

### 2.3 Statistical analysis

SPSS version 22.0 (IBM, Armonk, NY) and GraphPad Prism version 5.0 Software (San Diego, CA, USA) were used for data analysis and plotting. The measurement data in line with the normal distribution were expressed as mean ± standard deviation, the difference between 2 groups was compared using Independent Samples *t*-test, and the comparison among more than 2 groups was performed using analysis of variance. The difference in enumeration data was analyzed using Chi-Squared test. The comparison of non-normal data among 2 or more groups was performed using Mann–Whitney *U* or Kruskal–Wallis test. Kaplan–Meier method and log-rank test were used to analyze the relation between CBX8 expression and survival rate of patients with cervical cancer. Risk factors affecting the prognosis of patients with cervical cancer were further subjected to univariate and multivariate analyses. The receiver operating characteristic (ROC) curve was used to calculate the area under the curve of each index to compare the accuracy of each systemic inflammatory index to predict the prognosis. *P* < .05 was considered to be statistically significant.

## 3 Results

### 3.1 Chromobox homolog 8 was highly expressed in cervical cancer tissues

**Figure 1:**
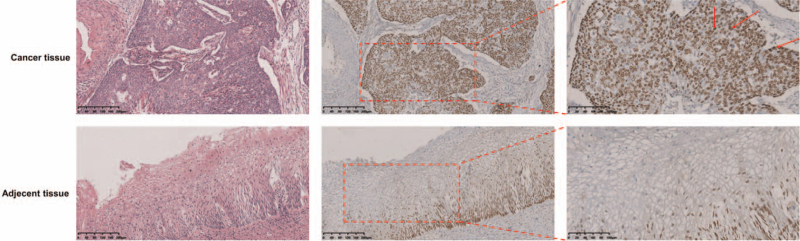
HE staining and CBX8 immunohistochemistry staining of cervical cancer tissue and adjacent tissue samples (original magnification, × 100 and × 200).

**Figure 2:**
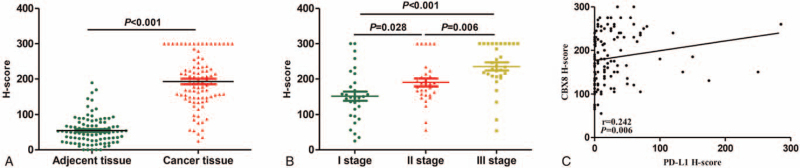
(A) H-score of CBX8 in paired cervical cancer samples and adjacent normal tissues (n = 93). (B) H-score of CBX8 in different FIGO stage. (C) The correlation between CBX8 and PD-L1 in 126 cervical cancer.

To study the expression of CBX8 in cervical cancer tissues, the cervical cancer and paracarcinoma tissue chips OD-CT-RpUtr03–004, OD-CT-RpUtr03–005, and OD-CT-RpUtr03–006 were used. OD-CT-RpUtr03–004 contained 31 pairs of cervical squamous cell carcinoma tissues and adjacent normal tissues. All the 31 patients had international federation of gynecology and obstetrics (FIGO) stage I cervical cancer. OD-CT-RpUtr03–005 contained 31 pairs of cervical squamous cell carcinoma tissues and adjacent normal tissues. All the 31 patients had FIGO stage II cervical cancer. OD-CT-RpUtr03–006 contained 31 pairs of cervical squamous cell carcinoma tissues and adjacent normal tissues. All the 31 patients had FIGO stage III cervical cancer. IHC results showed that CBX8 was located mainly in the nuclei of cervical cancer tissues (Fig. 1). H score semi-quantitative analysis showed that among 93 cases of cervical cancer, the expression of CBX8 (192.69 ± 73.78) was significantly higher in cervical cancer tissues than that in normal cervical tissues (54.80 ± 38.46), and the difference was statistically significant (*P* < .001) (Fig. 2A). In addition, it was confirmed that CBX8 had the highest expression in FIGO stage III (235.55 ± 63.84), followed by FIGO stage II (190.39 ± 61.07) and FIGO stage I (152.13 ± 72.72; all *P* < .001; Fig. 2B).

### 3.2 Relationship between chromobox homolog 8 expression and clinicopathology of cervical cancer

HUteS169Su01 chip contained 126 cases of cervical cancer tissues with complete clinicopathological data, PD-L1 expression, and follow-up data. The patients were aged 29 to 70 years old. In terms of FIGO stage, there were 69 cases of stage I, 30 cases of stage II, and 27 cases of stage III. There were 22 cases of pathological grade I, 26 cases of grade II, and 78 cases of grade III; 108 cases were HPV-positive and 18 cases were HPV-negative. The median follow-up time was 75 months (range: 8 to 86 months). The patients were divided into high- and low-expression groups with the H score 180 of median expression level of CBX8 as the cut-off value. The expression of CBX8 was significantly correlated with the FIGO stage and PD-L1 expression in patients with cervical cancer, and had no statistically significant difference in terms of the patient's age, HPV, lymphovascular invasion, pathological grade, and lymph node metastasis (*P* > .05; Table 1). We found that the expression of CBX8 was proportional to the FIGO stage (*P* < .05), which is consistent with the above results. In addition, Pearson test was used to analyze the correlation between CBX8 and PD-L1 expression, which showed that CBX8 and PD-L1 expression were significantly positively correlated with each other (*r* = 0.242; *P* = .006; Fig. 2C).

**Table 1:** Relationship between CBX8 expression and clinicopathologic features of patients with cervical cancer.

		CBX8			
Clinicopathological features	Patients (n = 126)	Low	High	χ^2^	*P* value
Age					
≤45	60	32	28	0.51	.476
>45	66	31	35		
HPV					
Negative	18	10	8	0.26	.611
Positive	108	53	55		
Lymphovascular invasion					
Negative	90	45	45	0.00	1.00
Positive	36	18	18		
Histological grade					
I	22	15	7	3.52	.172
II	26	12	14		
III	78	36	42		
Lymph nodes					
Negative	101	52	49	0.45	.503
Positive	25	11	14		
FIGO stage					
I	69	39	30	7.97	.019
II	30	17	13		
III	27	7	20		
PD-L1					
Negative	36	25	11	7.62	.006
Positive	90	38	52		

CBX8 = chromobox homolog 8, FIGO = international federation of gynecology and obstetrics, HPV = human papillomavirus, PD-L1 = programmed death ligand-1.

### 3.3 Analysis of prognostic value of chromobox homolog 8 in patients with cervical cancer

**Figure 3:**
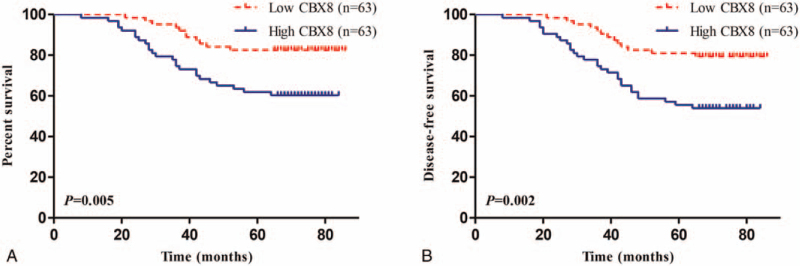
The overall survival (OS) (A) and disease-free survival (DFS) (B) of cervical cancer patients with high CBX8 expression was significantly poor than that of patients with low CBX8 expression.

Kaplan–Meier and log-rank tests were used for survival analysis to explore the prognostic value of CBX8 in patients with cervical cancer. Postoperative overall survival (OS) of patients with cervical cancer with high CBX8 expression was shorter (*P* = .005; Fig. 3A) than that of patients with low CBX8 expression, and the 5-year survival rate of the 2 groups was 60.3% and 82.5%, respectively. Postoperative disease-free survival (DFS) of patients with cervical cancer with high CBX8 expression was shorter (*P* = .002; Fig. 3B) than that of patients with low CBX8 expression. The 5-year DFS rate of the 2 groups was 54.0% and 79.4%, respectively. The univariate analysis results of clinicopathological characteristics and CBX8 expression levels that may affect the survival of patients with cervical cancer showed that patient age, lymphovascular invasion, lymph node metastasis, FIGO stage, and CBX8 expression level affected the OS and DFS of patients after surgery, and this difference was statistically significant (Tables 2 and 3). Subsequent multivariate analysis showed that patient age, lymphovascular invasion, FIGO stage, and CBX8 were independent prognostic factors for postoperative OS of patients with cervical cancer (Table 2). Patient age, FIGO stage, and CBX8 were independent prognostic factors of postoperative DFS in patients with cervical cancer (Table 3). Based on the above results, CBX8 may be an independent prognostic factor for patients with cervical cancer.

**Table 2:** Cox proportional hazards model univariate and multivariate analyses of overall survival for 126 cervical cancer patients.

	Univariate analysis		Multivariate analysis	
Variables	HR (95% CI)	*P* value	HR (95% CI)	*P* value
Age				
>45 yrs vs ≤45 yrs	7.33 (2.85–18.89)	<0.001^∗^	5.66 (2.11–15.17)	.001^∗^
HPV				
Positive vs Negative	1.49 (0.53–4.22)	0.452		
Lymphovascular invasion				
Positive vs Negative	4.66 (2.40–9.07)	<0.001^∗^	2.35 (1.13–4.88)	.022^∗^
Histological grade				
III vs. I-II	1.34 (0.93–1.93)	0.114		
Lymph nodes				
Positive vs. Negative	4.49 (2.32–8.69)	<0.001^∗^	1.00 (0.48–2.08)	.991
FIGO stage				
III-II vs. I	14.09 (4.97–39.95)	<0.001^∗^	11.08 (3.66–33.54)	<.001^∗^
PD-L1				
High vs Low	1.67 (0.82–3.40)	0.156		
CBX8				
High vs Low	2.68 (1.32–5.45)	0.006^∗^	2.47 (1.19–5.15)	.016^∗^

CBX8 = chromobox homolog 8, FIGO = international federation of gynecology and obstetrics, HPV = human papillomavirus, PD-L1 = programmed death ligand-1.

**Table 3:** Cox proportional hazards model univariate and multivariate analyzes of disease-free survival for 126 cervical cancer patients.

	Univariate analysis		Multivariate analysis	
Variables	HR (95% CI)	*P* value	HR (95% CI)	*P* value
Age				
>45 yrs vs ≤45 yrs	5.22 (2.41–11.29)	<.001^∗^	4.31 (1.93–9.61)	<.001^∗^
HPV				
Positive vs Negative	1.81 (0.65–5.08)	.259		
Lymphovascular invasion				
Positive vs Negative	3.36 (1.83–6.16)	<.001^∗^	1.89 (0.97–3.68)	.060
Histological grade				
III vs. I-II	1.32 (0.95–1.85)	.101		
Lymph nodes				
Positive vs Negative	3.54 (1.89–6.61)	<.001^∗^	1.11 (0.55–2.23)	.766
FIGO stage				
III-II vs I	9.29 (4.11–20.99)	<.001^∗^	8.00 (3.33–19.23)	<.001^∗^
PD-L1				
High vs Low	1.29 (0.69–2.43)	.428		
CBX8				
High vs Low	2.69 (1.40–5.19)	.003^∗^	2.50 (1.28–4.89)	.007^∗^

CBX8 = chromobox homolog 8, FIGO = international federation of gynecology and obstetrics, HPV = human papillomavirus, PD-L1 = programmed death ligand-1.

### 3.4 Prognostic value of chromobox homolog 8 and programmed death ligand-1 in patients with cervical cancer

**Figure 4:**
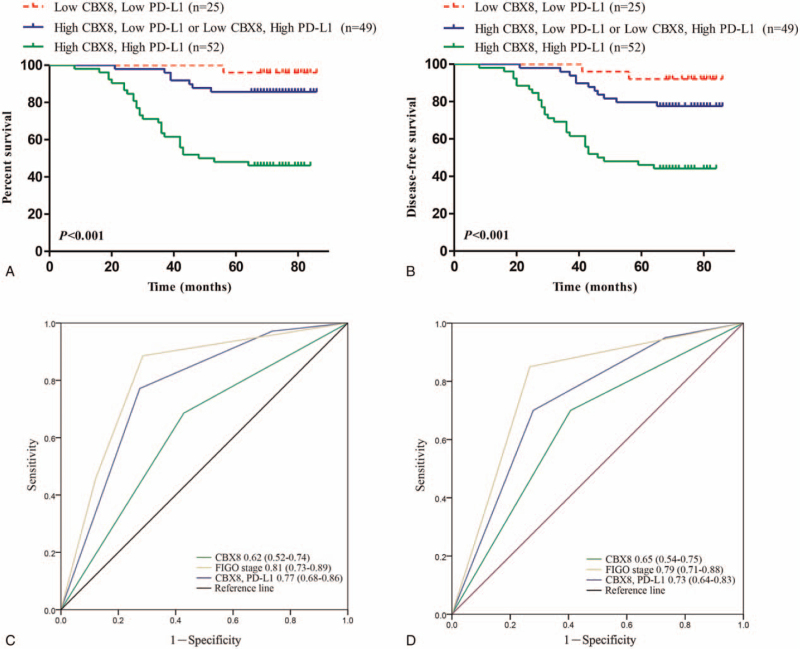
The overall survival (OS) (A) and disease-free survival (DFS) (B) of cervical cancer patients with high expression of CBX8 and PD-L1 was significantly poor than other patients. The prognostic ability of CBX8 and PD-L1 co-expression model was compared with CBX8 and FIGO stage based on OS (C) and DFS (D) by ROC in 5-years.

To investigate whether the co-expression of CBX8 and PD-L1 can be used to predict the prognostic value for patients with cervical cancer, the patients were divided into 3 groups based on the expression of CBX8 and PD-L1: those with low expression of CBX8 and PD-L1, those with high expression of CBX8 and low expression of PD-L1 or low expression of CBX8 and high expression of PD-L1, and those with high expression of CBX8 and PD-L1. Kaplan–Meier analysis revealed that the survival status of these 3 groups of patients differed. Cervical cancer patients with high expression of CBX8 and PD-L1 have a poor prognosis (five-year survival rate of only 46.2%), while cervical cancer patients with low expression of CBX8 and PD-L1 have a better prognosis (a five-year survival rate of 96%) (Fig. 4A). Similar results were obtained for DFS (Fig. 4B). In addition, the prognostic value of CBX8 and PD-L1 co-expression model was analyzed by ROC and compared with CBX8 and FIGO stage. It was found that the area under the curve of the CBX8 and PD-L1 co-expression model was significantly higher than that of CBX8, and similar to that of FIGO stage in 5 years (Fig. 4C, D), indicating that CBX8 and PD-L1 co-expression model can accurately predict the postoperative prognosis of patients with cervical cancer.

## 4 Discussion

As the fourth most common female malignant tumor in the world, cervical cancer is a major challenge to global health.^[1]^ Among the 270,000 cervical cancer deaths in 2015, approximately 90% occurred in low- and middle-income countries, whose death rate was 18 times that in developed countries.^[3]^ Two high-risk subtypes of HPV almost cause all cervical cancers. HPV screening and vaccination programs are effective preventive strategies for disease.^[14]^ Although progress has been made in the prevention, screening, diagnosis, and treatment of cervical cancer in the past decade, the treatment results of cervical cancer greatly vary among regions in the world.^[15]^

CBX8 is an important member of PcG family, which can regulate certain genes to inhibit transcription and translation by modifying his tones and chromatin. The *CBX8* protein-coding gene is located in chromosome 17. Its protein is mainly expressed in the nucleus, participates in the modification and transport of the Golgi apparatus, binding single-stranded RNA, SUMOylation, and ubiquitin protein transport enzyme activity, and is involved in maintaining the transcriptional inhibition and expression of many genes.^[16]^ As a transcription inhibitor, CBX8 mainly inhibits cell proliferation through INK4α-ARF-dependent and -independent pathways. In recent years, studies have shown that CBX8 expression imbalance is closely related to the occurrence, development, treatment, and prognosis of malignant tumors. Zhang et al found that CBX8 is highly expressed in hepatocellular carcinoma (HCC), and the prognosis of patients with HCC with high CBX8 expression is poor. Furthermore, a mechanism study confirmed that CBX8 directly binds to the EGR1 promoter, thus activating the AKT/β-catenin signaling pathway.^[17]^ Gao et al found that the expression levels of CBX8 and BMI-1 is substantially higher in HCC tissues. Kaplan–Meier survival curve analysis showed that the 5-year OS and DFS of patients with high expression of CBX8 and BMI1 are shorter than those of patients with low expression of CBX8 and BMI1.^[12,18]^ Yang et al found that insulin growth factor-1 can promote the proliferation of colon cancer cells by promoting the expression of CBX8.^[19]^ Xiao et al. found that the expression of CBX8 in esophageal cancer tissues is higher than that in the corresponding tissues adjacent to the tumor, significantly related to the tumor, node, metastasis stage of esophageal cancer, and is upregulated after radiotherapy and chemotherapy. In vitro experiments have demonstrated that knockout of CBX8 can inhibit the clone formation of esophageal cancer cells, thus reducing the size and weight of transplanted tumors in nude mice. Downregulating CBX8 can upregulate the expression of P21, CHK1, and WEE1, thereby inhibiting cyclin-dependent kinases, prolonging the cell cycle, and reducing cell proliferation.^[20]^ Chung et al demonstrated that CBX8 is the main regulator of breast cancer in vivo and in vitro, and its expression is upregulated in breast cancer tissues, which indicates poor prognosis of patients with breast cancer. Transfection with short hairpin RNA in breast cancer cell lines to knock down CBX8 leads to inhibition of the clone-formation ability of cells, and reduction in the volume and weight of transplanted tumors in nude mice. It has been found in mechanism studies that CBX8 is associated with non-Pc repressive complex-1, including the H3K4 methyltransferase–Wdr5 complex, and they jointly regulate the expression of *NOTCH*.^[21]^ Tan et al showed that the chromosomal translocation of the mixed lineage leukemia (MLL) gene leads to the occurrence of acute leukemia, and CBX8 plays an important role in the transcriptional regulation of MLL-AF9. CBX8 interacts with the C-terminal domain of MLL-AF9 to mediate the transcriptional activation of the target gene of fusion protein MLL-AF9, thereby inducing leukemia.^[8]^ The above findings demonstrate that CBX8 mainly plays a key role as an oncogene in some tumors. In addition, studies have shown that CBX8 plays a contradictory role in tumors. Wang et al. identified that CBX8 can promote the proliferation of esophageal squamous cell carcinoma, but inhibit its metastasis as well.^[13]^ Tang et al found similar results in colorectal cancer that CBX8 can promote cell proliferation, but inhibit cell metastasis and invasion.^[22]^

Although the prognostic value of CBX8 and PD-L1 co-expression in cervical cancer was demonstrated, the study had a few limitations. Firstly, the expression of CBX8 was detected using tissue chips of Shanghai Outdo Biotech, and more patient information such as postoperative treatment plan could not be obtained. Secondly, IHC analysis alone may not be sufficient to accurately explain the role of CBX8 in cervical cancer. Thirdly, the reason why co-expression of CBX8 and PD-L1 could predict the prognosis of cervical cancer patients was not explored. Therefore, the prognostic significance of CBX8 in cervical cancer remains to be further verified by large-scale prospective studies. In addition, molecular and cellular research should be performed in the future to comprehensively analyze the mechanism of action of CBX8, and further explore and clarify the role of its target genes.

In this study, tissue chips were used for IHC. It was found that CBX8 was highly expressed in cervical cancer tissues, and the expression of CBX8 was closely related to FIGO stage. The higher the stage, the higher the expression of CBX8. The DFS and OS of cervical cancer patients with high CBX8 expression were significantly shorter than those of patients with low CBX8 expression. CBX8 was an independent prognostic factor for cervical cancer patients. In addition, CBX8 and PD-L1 co-expression model could be used to better predict the prognosis of cervical cancer patients, and the prediction results were similar to those of FIGO stage.

## 5 Conclusion

CBX8 is highly expressed in cervical cancer, positively correlated with PD-L1 expression, and significantly correlated with FIGO stage. DFS and OS of patients with cervical cancer with high CBX8 expression were found to be significantly shorter than those of patients with low CBX8 expression. Thus, CBX8 may be an independent prognostic factor for patients with cervical cancer. In addition, CBX8 and PD-L1 co-expression model could effectively predict the postoperative survival of patients with cervical cancer, which may aid medical decisions in clinical settings.

## Author contributions

**Conceptualization:** Mingcai Shui.

**Data curation:** Hui Zhou.

**Formal analysis:** Anhong Li.

**Funding acquisition:** Mingcai Shui.

**Investigation:** Chuan Li.

**Resources:** Chuan Li.

**Software:** Anhong Li.

**Supervision:** Mingyong Wu.

**Validation:** Mingyong Wu, Dan Jin.

**Writing – original draft:** Hui Zhou.

**Writing – review & editing:** Dan Jin.

## References

[1] Bray F Ferlay J Soerjomataram I Siegel RL Torre LA Jemal A. Global cancer statistics 2018: GLOBOCAN estimates of incidence and mortality worldwide for 36 cancers in 185 countries. CA Cancer J Clin 2018;68:394–424. 30207593 10.3322/caac.21492

[2] Cohen PA Jhingran A Oaknin A Denny L. Cervical cancer. Lancet 2019;393:169–82. 30638582 10.1016/S0140-6736(18)32470-X

[3] Vaccarella S Lortet-Tieulent J Plummer M Franceschi S Bray F. Worldwide trends in cervical cancer incidence: impact of screening against changes in disease risk factors. Eur J Cancer 2013;49:3262–73. 23751569 10.1016/j.ejca.2013.04.024

[4] Chen W Zheng R Baade PD. Cancer statistics in China, 2015. CA Cancer J Clin 2016;66:115–32. 26808342 10.3322/caac.21338

[5] Fokom Domgue J Schmeler KM. Conservative management of cervical cancer: current status and obstetrical implications. Best Pract Res Clin Obstet Gynaecol 2019;55:79–92. 30029960 10.1016/j.bpobgyn.2018.06.009

[6] Lewis EB. A gene complex controlling segmentation in Drosophila. Nature 1978;276:565–70. 103000 10.1038/276565a0

[7] Kerppola TK. Polycomb group complexes--many combinations, many functions. Trends Cell Biol 2009;19:692–704. 19889541 10.1016/j.tcb.2009.10.001 PMC3038206

[8] Tan J Jones M Koseki H. CBX8, a polycomb group protein, is essential for MLL-AF9-induced leukemogenesis. Cancer Cell 2011;20:563–75. 22094252 10.1016/j.ccr.2011.09.008 PMC3220883

[9] Lee SH Um SJ Kim EJ. CBX8 suppresses Sirtinol-induced premature senescence in human breast cancer cells via cooperation with SIRT1. Cancer Lett 2013;335:397–403. 23474493 10.1016/j.canlet.2013.02.051

[10] Cornwall JIMS. Remarks on the vaccine treatment of colicystitis. Ind Med Gaz 1908;43:82–5. 29006512 PMC5182984

[11] Song X Tang T Li C Liu X Zhou L. CBX8 and CD96 are important prognostic biomarkers of colorectal cancer. Med Sci Monit 2018;24:7820–7. 30383736 10.12659/MSM.908656 PMC6225733

[12] Tang B Tian Y Liao Y. CBX8 exhibits oncogenic properties and serves as a prognostic factor in hepatocellular carcinoma. Cell Death Dis 2019;10:52. 30718464 10.1038/s41419-018-1288-0 PMC6361915

[13] Wang G Tang J Zhan W. CBX8 suppresses tumor metastasis via repressing snail in esophageal squamous cell carcinoma. Theranostics 2017;7:3478–88. 28912889 10.7150/thno.20717 PMC5596437

[14] Crosbie EJ Einstein MH Franceschi S Kitchener HC. Human papillomavirus and cervical cancer. Lancet 2013;382:889–99. 23618600 10.1016/S0140-6736(13)60022-7

[15] Cibula D Potter R Planchamp F. The European Society of Gynaecological Oncology/European Society for Radiotherapy and Oncology/European Society of Pathology Guidelines for the Management of Patients with Cervical Cancer. Virchows Arch 2018;472:919–36. 29725757 10.1007/s00428-018-2362-9

[16] Vandamme J Volkel P Rosnoblet C Le Faou P Angrand PO. Interaction proteomics analysis of polycomb proteins defines distinct PRC1 complexes in mammalian cells. Mol Cell Proteomics 2011;10:M110002642. 10.1074/mcp.M110.002642 PMC3069339 21282530

[17] Zhang CZ Chen SL Wang CH. CBX8 exhibits oncogenic activity via AKT/beta-catenin activation in hepatocellular carcinoma. Cancer Res 2018;78:51–63. 29066512 10.1158/0008-5472.CAN-17-0700

[18] Gao SB Sun SL Zheng QL. Genetic alteration and misexpression of Polycomb group genes in hepatocellular carcinoma. Am J Cancer Res 2015;5:2969–79. 26693053 PMC4656724

[19] Yang S Liu W Li M Wen J Zhu M Xu S. Insulin-like growth factor-1 modulates polycomb Cbx8 expression and inhibits colon cancer cell apoptosis. Cell Biochem Biophys 2015;71:1503–7. 25398592 10.1007/s12013-014-0373-y

[20] Xiao W Ou C Qin J. CBX8, a novel DNA repair protein, promotes tumorigenesis in human esophageal carcinoma. Int J Clin Exp Pathol 2014;7:4817–26. 25197352 PMC4152042

[21] Chung CY Sun Z Mullokandov G. Cbx8 acts non-canonically with wdr5 to promote mammary tumorigenesis. Cell Rep 2016;16:472–86. 27346354 10.1016/j.celrep.2016.06.002 PMC4972459

[22] Tang J Wang G Zhang M. Paradoxical role of CBX8 in proliferation and metastasis of colorectal cancer. Oncotarget 2014;5:10778–90. 25360999 10.18632/oncotarget.2502 PMC4279409

